# Enhanced itaconic acid production in *Aspergillus niger* using genetic modification and medium optimization

**DOI:** 10.1186/1472-6750-12-57

**Published:** 2012-08-27

**Authors:** An Li, Nina Pfelzer, Robbert Zuijderwijk, Peter Punt

**Affiliations:** 1 TNO Microbiology and Systems biology, PO Box 360, 3700 AJ Zeist, The Netherlands; 2 Molecular Microbiology & Biotechnology Leiden University, Universiteit Leiden, Postbus 9500, 2300 RA, Leiden, Netherlands

## Abstract

**Background:**

*Aspergillus niger* was selected as a host for producing itaconic acid due to its versatile and tolerant character in various growth environments, and its extremely high capacity of accumulating the precursor of itaconic acid: citric acid. Expressing the CAD gene from *Aspergillus terreus* opened the metabolic pathway towards itaconic acid in *A. niger*. In order to increase the production level, we continued by modifying its genome and optimizing cultivation media.

**Results:**

Based on the results of previous transcriptomics studies and research from other groups, two genes : *gpdA* encoding the glyceraldehyde −3-dehydrogenase (GPD) and *hbd1* encoding a flavohemoglobin domain (HBD) were overexpressed in *A. niger*. Besides, new media were designed based on a reference medium for *A. terreus*. To analyze large numbers of cultures, we developed an approach for screening both fungal transformants and various media in 96-well micro-titer plates. The *hbd1* transformants (HBD 2.2/2.5) did not improve itaconic acid titer while the *gpdA* transformant (GPD 4.3) decreased the itaconic acid production. Using 20 different media, copper was discovered to have a positive influence on itaconic acid production. Effects observed in the micro-titer plate screening were confirmed in controlled batch fermentation.

**Conclusions:**

The performance of *gpdA* and *hbd1* transformants was found not to be beneficial for itaconic acid production using the tested cultivation conditions. Medium optimization showed that, copper was positively correlated with improved itaconic acid production. Interestingly, the optimal conditions for itaconic acid clearly differ from conditions optimal for citric- and oxalic acid production.

## Background

Itaconic acid is a white crystalline unsaturated C5 dicarboxylic acid. Because of its specific favorable properties and the unique structure, itaconic acid is used worldwide as monomer or co-monomer in manufacturing plastics, resins etc [1,2]. Its market price is around 2 US$ per kilo [3]. Currently, itaconic acid is produced commercially by *Aspergillus terreus* (*A. terreus*) via submerged fungal fermentations [3]. In a transcriptomics study in *A. terreus*, several itaconic acid-related genes were identified [4]. Among them, *cadA* encoding *cis*-aconitate decarboxylase (CAD) which is the key enzyme for itaconic acid production from citric acid in the tri-carboxylic acid (TCA) cycle. Based on its high citric acid producing capability and broad applicability in industry, *Aspergillus niger* (*A. niger*) was selected as a novel itaconic acid production host strain in recent work. Expression of *cadA* in *A. niger* leads to itaconic acid production in *A. niger*[4]. In our attempts to enhance itaconic acid production levels in *A. niger* CAD strains we used genetic modification of the production host and medium optimization.

A first target to enhance itaconic acid production in *A. niger* CAD transformants is glyceraldehyde-3-phosphate dehydrogenase (GPD)*.* GPD, encoded by *gpdA* is a key enzyme in glycolysis which converts glyceraldehyde-3-phosphate into biphosphoglycerate [5]. In our previous transcriptomics analysis, this gene is highly expressed under itaconic acid production conditions [4] and its function in biological pathway may indicate a role in generating an increased flux through glycolysis towards the TCA cycle [5]. The relevance of increased glycolytic flux in itaconic acid production is also suggested by the results of Tevž and co-workers [6]. They show that overexpression of a modified *pfkA* gene (encoding 6-phosphofructo-1-kinase) from *A. niger* in *A. terreus* results in enhanced itaconic acid production, probably due to increased glycolytic flux*.* Therefore, over-expression of *gpdA* in *A. niger* CAD strains might increase itaconic acid producing level via enhanced glycolytic flux.

The second target to enhance itaconic acid production in *A. niger* CAD transformants is Favohemoglobin (Fhb), as heterologous Fhb overexpression has been shown to enhance itaconic acid production in *A terreus*[7]. In our study, over-expression of fungal hemoglobin domain (HBD, encoded by *hbd1*) in *A. niger* is used as a potential enhancer of itaconic acid production.

In addition to genetic modification of *A. niger*, we also focused on development of an itaconic acid production medium for *Aspergillus niger*. Medium optimization for *A. niger* and *A. terreus* has been carried out over the last 40 years. Media for citric acid and itaconic acid production were accomplished by various research groups [8-13]. The required medium components for these two organic acids are quite comparable: high concentration of glucose (7.5-15 %) and magnesium sulfate, low nitrogen and phosphorus, low but adequate levels of zinc, copper and iron, limited manganese (around 10 ppb). Since the existing industrial itaconic acid production medium for *A. terreus* has not yet been adapted to *A. niger*, we aimed for an optimized itaconic acid production medium for *A. niger*. As citric acid is the potential substrate of itaconic acid in *A. niger*, in our medium design the composition of citric acid production medium was considered as well.

## Methods

### Strains /plasmids /Fungal transformation

The uridine auxotrophic *A. niger* strains AB 1.13 and AB 1.13 CAD 10.1 pyrG- which was derived from AB 1.13 via *cadA* insertion [4] were used as parental strain for *A. niger* transformation. The plasmid used for overexpressing *gpdA* contained *a 5.5 kb Pst*I *A. niger* fragment, including the *gpdA* gene, in pUC19 (pAB 5–1 [14]). The plasmid for overexpression of *hbd1* (pHBN) was previously described [15]. The plasmid pAB 4.1 [16] containing the pyrG + gene was used as a selection marker for co-transformation. To over-express *gpdA* and *hbd1* in the *A. niger* CAD strain, the plasmids pAB 5–1 and pHBN were co- transformed with pAB4-1 respectively. All transformants were further selected for their ability to growth on Minimal Medium plates (Vogel’s medium) without uridine.

### A. niger *transformants screening*

For screening and selection of AB 1.13 CAD + GPD and HBD transformants, 96-wells micro-titer plates (Costar® 3799, round bottom with lid) were filled with *A. niger* cultivation medium, 250 μl medium per well. The inner 60 wells were inoculated with spores from target strains, using sterilized tooth picks. The outermost wells of the plate were kept empty to prevent the effect of evaporation. In order to prevent contamination, each cultivation plate was sealed with a sterilized oxygen permeable film directly after spore inoculation. At the end of cultivation (72 hours for the first batch and 50 hours for the second batch), cultures were harvested and used for HPLC analysis.

### Medium design and culture conditions

Based on the reference medium (Medium 1[4], Table 1) , 19 new media were generated by differing one component or its amount compared to the reference medium (Table 2). To find changes compared to the reference medium, the amount of the differing component was either the maximal or the minimal value of the one used in literature before. To generate a suitable medium for itaconic acid production in *A. niger* transformants, 20 media and including the reference were analyzed in triplicate. To prevent cross contamination, each plate was inoculated with only one transformant. For further media improvement, media which showed a positive effect on itaconic acid production were combined and concentration ranges were extended. 

**Table 1:** **Composition of the 20 cultivation media. 1 is the reference medium of itaconic acid production by*A. terreus***

	**1**	**2**	**3**	**4**	**5**	**6**	**7**	**8**	**9**	**10**
	**g/L**	**g/L**	**g/L**	**g/L**	**g/L**	**g/L**	**g/L**	**g/L**	**g/L**	**g/L**
Glucose	100	50	140	100	100	100	100	100	100	100
NaNO3	-	-	-	0.5	2.4	-	-	-	-	-
(NH4)2SO4	2.36	2.36	2.36	-	-	-	-	2.36	2.36	2.36
NH4NO3	-	-	-	-	-	3.1	5	-	-	-
KH2PO4	0.11	0.11	0.11	0.11	0.11	0.11	0.11	3	0.11	0.11
MgSO4 x 7 H2O	0.5	0.5	0.5	0.5	0.5	0.5	0.5	0.5	0.24	2.2
CuSO4 * 5 H2O	0.0002	0.0002	0.0002	0.0002	0.0002	0.0002	0.0002	0.0002	0.0002	0.0002
FeIIISO4 * 7 H2O	0.0055	0.0006	0.0006	0.0006	0.0006	0.0006	0.0006	0.0006	0.0006	0.0006
FeCl3	-	-	-	-	-	-	-	-	-	-
MnCl2 * 4 H2O	0.0007	-	-	-	-	-	-	-	-	-
ZnSO4 * 7 H2O	0.0013	0.0006	0.0006	0.0006	0.0006	0.0006	0.0006	0.0006	0.0006	0.0006
NiCl2 * 6 H2O	-	-	-	-	-	-	-	-	-	-
KCl	-	-	-	-	-	-	-	-	-	-
NaCl	0.074	0.074	0.074	0.074	0.074	0.074	0.074	0.074	0.074	0.074
CaCl2 * 2 H2O	0.13	0.13	0.13	0.13	0.13	0.13	0.13	0.13	0.13	0.13
Uridine	2.45	2.45	2.45	2.45	2.45	2.45	2.45	2.45	2.45	2.45
	**11**	**12**	**13**	**14**	**15**	**16**	**17**	**18**	**19**	**20**
	**g/L**	**g/L**	**g/L**	**g/L**	**g/L**	**g/L**	**g/L**	**g/L**	**g/L**	**g/L**
Glucose	100	100	100	100	100	100	100	100	100	100
NaNO3	-	-	-	-	-	-	-	-	-	2.4
NH4SO4	2.36	2.36	2.36	2.36	2.36	2.36	2.36	2.36	2.36	-
NH4NO3	-	-	-	-	-	-	-	-	-	-
KH2PO4	0.11	0.11	0.11	0.11	0.11	0.11	0.11	0.11	0.11	0.11
MgSO4 x 7 H2O	0.5	0.5	0.5	0.5	0.5	0.5	0.5	0.5	0.5	0.5
CuSO4 * 5 H2O	-	0.0025	0.0002	0.0002	0.0002	0.0002	0.0002	0.0002	0.0002	0.0002
FeIIISO4 * 7 H2O	0.0006	0.0006	0.0138	-	-	0.0006	0.0006	0.0006	0.0006	0.0006
FeCl3	-	-	-	0.0006	0.0001	-	-	-	-	-
MnCl2 * 4 H2O	-	-	-	-	-	0.007	-	-	-	-
ZnSO4 * 7 H2O	0.0006	0.0006	0.0006	0.0006	0.0006	0.0006	0.022	0.0006	0.0006	0.0006
NiCl2 * 6 H2O	-	-	-	-	-	-	-	0.0005	-	-
KCl	-	-	-	-	-	-	-	-	-	0.52
NaCl	0.074	0.074	0.074	0.074	0.074	0.074	0.074	0.074	2	-
CaCl2 * 2 H2O	0.13	0.13	0.13	0.13	0.13	0.13	0.13	0.13	0.2	-
Uridine	2.45	2.45	2.45	2.45	2.45	2.45	2.45	2.45	2.45	2.45

**Table 2:** Media components and their ranges from literature studies used for optimization

**Media optimization**		**Value**		**reference**
**Source**		***min (g/L)***	***max (g/L)***	
C	Glucose	50	140	[9,10,13]
N	NaNO3	0.5	2.4	[4]
	NH4SO4	2.36	2.36	[27]
	NH4NO3	3.1	5	[9,10]
P	KH2PO4	0.11	3	[4,27]
Mg	MgSO4 x 7 H2O	0.24	2.2	[4,9]
Cu	CuSO4 x 5 H2O	0	0.0025	[4]
Fe	Fe2SO4 x 7 H2O	0.0006	0.0138	[4,28]
	FeCl3	0.0006	0.0001	[4,9]
Mn	MnCl2 x 4 H2O	0	0.007	[11,27]
Zn	ZnSO4 x 7 H2O	0.0006	0.022	[12]
Ni	NiCl2 x 6 H2O	0	0.0005	[4]
K	KCl	0.52	0.52	[4]
	NaCl	0,074	2	[4,27]
	CaCl2 * 2H2O	0,13	0,2	[4,27]

After seeding, all plates were directly sealed with an oxygen permeable film (Sealing film sterile, breathable M20193, Dispolab the Netherlands), placed in a plastic air bag and cultivated in a 33 °C, 850 rpm incubator (Microtron, Infos-ht) for 72 hours (first batch) or 50 hours (second batch).

Controlled batch fermentations were carried out as follows: Media were prepared in demineralized water. Pre-cultures of 100 ml (10^6^ spores/ml) in 500 ml baffled Erlenmeyer flasks were inoculated for two days. As described previously [4], fermentations were carried out in 5 L Benchtop Fermentors (BioFlo 3000, New Brunswick Scientific Co., Inc.), at 33 degree. Air was used for sparging the bioreactor at a constant flow of 0.25 vvm (vol. gas (vol. liquid^-1^ min^-1^), dissolved oxygen tension (D.O.) was maintained minimal at 25 % coupled with stirred speed of 400–1000 rpm, pH was initiated at 3.5 adjusted by 1.5 M H_3_PO_4_ (acid) and kept at 2.3 by addition of 4 M KOH (base). Struktol (Schill and Seilacher) was applied as antifoam agent though-out the fermentation.

### Metabolite analysis

For metabolite analysis, 100 μl medium was pipetted from each culture to total recovery vials for High performance liquid chromatography (HPLC) analysis. Two analysis detection facilities were applied. The one from Waters Cooperate an 87 H-Aminex organic acids column (Bio-red), with 0.5 M H_2_SO_4_ as eluent and a Photodiode Array (PDA) detector for organic acids and Refractive Index (RI) detector for sugar like compounds. The other from Thermo Fisher (Dionex ICS 3000), analyzing organic acids through an organic acids column IonPac® ICE AS6, with 1.6 mM Heptafluorobutyric acid as eluent and a detector of suppressed conductivity CD25. Standard compounds (oxalic acid, gluconic acid, citric acid, *cis*-aconitic acid and itaconic acid) with concentrations of 100 mg/L, 200 mg/L, 500 g/L, 750 mg/L, 1000 mg/L and 2000 mg/L were used for calibration.

## Results

### Host strain selection and modification

**Figure 1:**
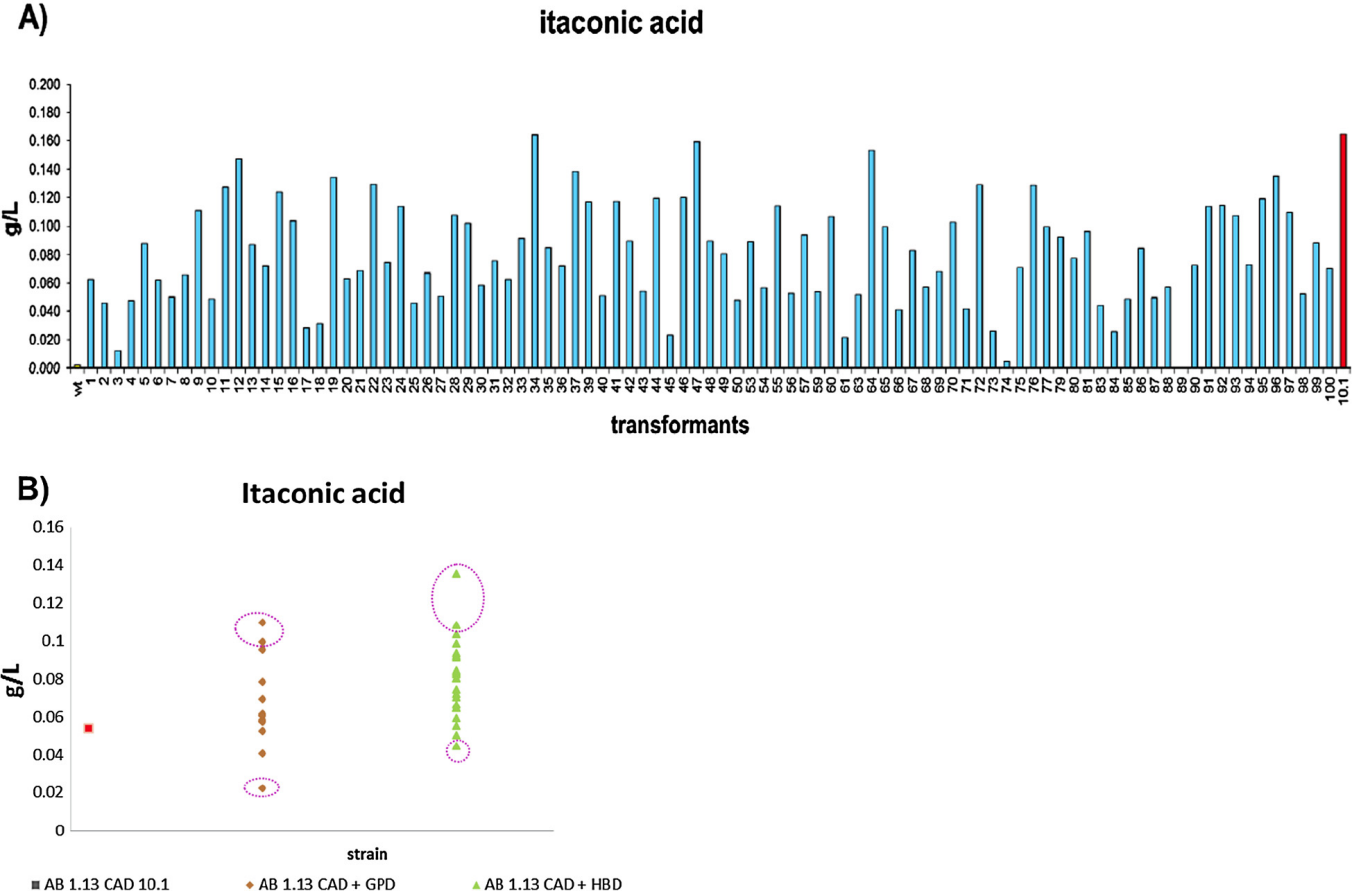
**Itaconic acid production of *A. niger *transformants. A **): Itaconic acid HPLC analysis of micro titer plate screening of *A. niger* CAD transformants. **B**): Itaconic acid screening results in micro-titer plate for the selected GPD and HBD transformants. The parental strain CAD 10.1 was used as a control. The transformants with the highest production and lowest production surrounded by an oval circle were selected further for Southern blot analysis.

The itaconic acid producing strain AB 1.13 CAD 10.1 pyrG- (CAD 10.1) was selected from a limited number of *cadA* transformants [4]. In an attempt to isolate a further improved itaconic acid producing strains, new transformants were generated. More than 90 colonies were screened for their itaconic acid production in a 96 well micro-titer plate together with CAD 10.1 as a reference. As presented in Figure 1A, strain CAD 10.1 is shown to be among the best itaconic acid producing strains. Therefore, this strain was used for further genetic modification. 

To improve itaconic acid production level, the glyceraldehyde dehydrogenase gene (*gpdA*) and the gene encoding a fungal hemoglobin domain (*hbd1*) were co-transformed with the pyrG + marker into the selected host AB 1.13 CAD 10.1. To select improved itaconic acid-producing co-transformants, 14 *gpdA* and 23 *hbd1* transformants were screened in 96 well micro-titer plate using reference medium [4]. Three strains from each HBD and GPD differing most strongly from CAD 10.1 were selected and analyzed for the presence for extra *hbd1* or *gpdA* gene copies by Southern Blot analysis (Figure 1B).

Two of the three selected *hbd1* strains (HBD 2.2, HBD 2.5) with improved itaconic acid titers contained one (extra) *hbd1* gene copies compared to CAD 10.1. Unexpectedly, only strain GPD 4.3 which actually has lower itaconic acid titer is a true *gpdA* transformant (containing 4 extra gene copies), the other two strains are just pyrG + transformants from CAD10.1. We selected the two *hbd1* co-transformants (HBD 2.2/2.5), the *gpdA* co-transformant (GPD 4.3) and two pyrG + transformants of CAD 10.1 (pyr + 4.1/4.14) for further analysis of different cultivation media.

### Culture medium design

To optimize the production medium, 19 new media were generated based on the reference medium from *A. terreus*[4], by varying several components or their concentrations per medium (Tables2,1). To be able to compare many transformants and media components in one batch, a micro-titer plate screening was used.

**Figure 2:**
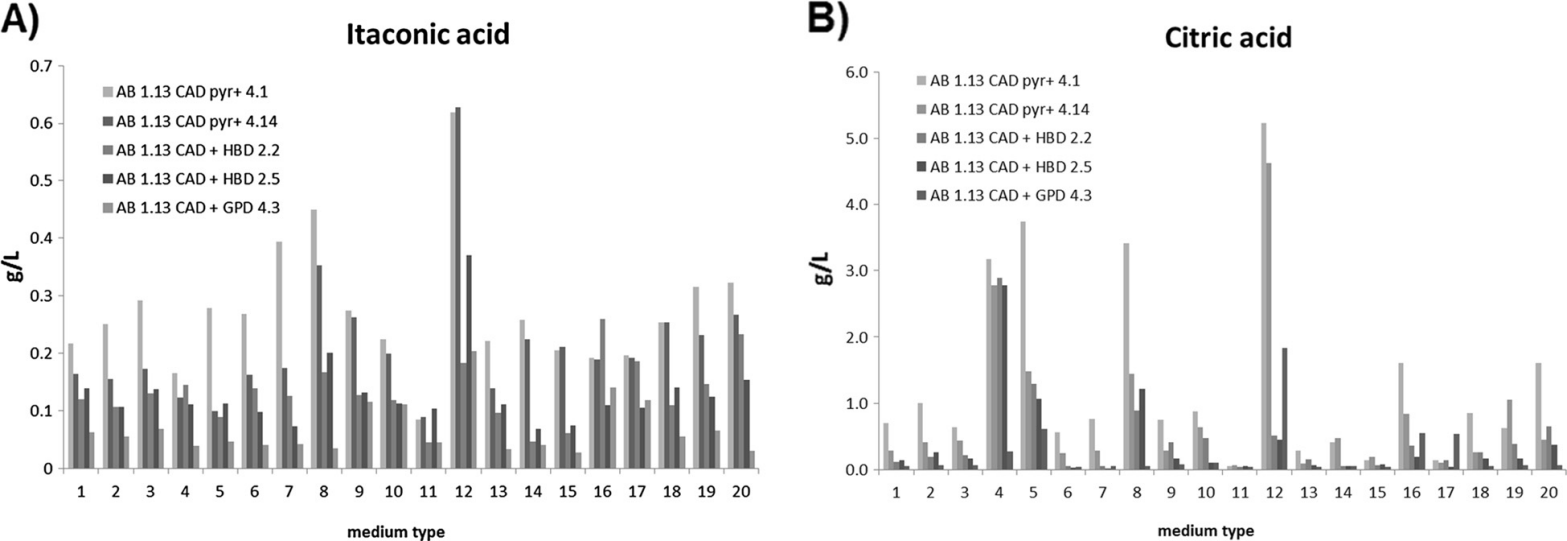
**Itaconic acid and citric acid production of *A. niger *in different media.** Itaconic acid (2**A**) and Citric acid (2**B**) level in micro-titer screening cultures using 20 different media. Standard deviations were less than 6 %.

After cultivation for about 70 hours, cultures from each medium were analyzed for itaconic acid production using HPLC. As indicated in Figure 2A, in comparison to reference medium 1, all strains showed increased itaconic acid production in Medium 12. In this medium, both pyrG + strains have even higher amount of itaconic acid production than the *hbd1* and *gpdA* strains (Figure 2A). In contrast, medium 11 and 13 yield the lowest itaconic acid production. Moreover, transformants grown in medium 8 and 20 also show improved itaconic acid production compared to the reference, except for transformant GPD 4.3. GPD 4.3 has generally low production level compared to other strains through all 20 media. The itaconic acid inducing components in the best media are phosphate (M8), copper (M12) and potassium (M20). The production pattern of citric acid is quite similar to itaconic acid in medium 8, 11, 12 and 13 (Figure 2B). In contrast, medium 4 provided high citric acid but low itaconic acid production. Medium 7 and 19 showed improved itaconic acid production only to the pyrG + strains. Based on the obtained results, we have retested a subset of the above mentioned media (8, 11, 12, 13, 20) and a newly designed medium (M20+) which combined the potentially itaconic acid inducing attributes (phosphorus 3 g/L, copper 0.0025 g/L and potassium 0.52 g/L) from medium 8, 12 and 20. Results are shown in Table 3. This time, strain FHB 2.5 was tested together with the reference strain pyr + 4.1 in triplicate. Similar results were obtained as in the initial screen. Medium 12 still yields the best itaconic acid production, whereas M20+ did not further improve production. In total, the standard deviations of the micro-titer plate screening results were less than 6 %. No oxalic acid was produced in the micro-titer plate cultivations.

**Table 3:** Itaconic acid level in micro-titer screening culture of strain AB 1.13 CAD + GPD 4.3 and AB 1.13 CAD + FHB 2.5 with media 1, 8, 11, 12, 13, 20 and 20+ (20+: combined medium of 8, 12 and 20)

	**GPD 4.3**	**FHB 2.5**
***Medium***	***Itaconic acid (g/L)***	***Itaconic acid (g/L)***
**1**	0.134 (±0.014)	0.134 (±0.025)
**8**	0.238 (±0.000)	0.249 (±0.059)
**11**	0.127 (±0.017)	0.072 (±0.032)
**12**	**0.260 (±0.016)**	**0.289 (±0.050)**
**13**	0.168 (±0.011)	0.076 (±0.020)
**20**	0.237 (±0.001)	0.218 (±0.028)
**20+**	0.080 (±0.011)	0.230 (±0.020)

### Effect of copper on organic acids production in batch fermentation

**Figure 3:**
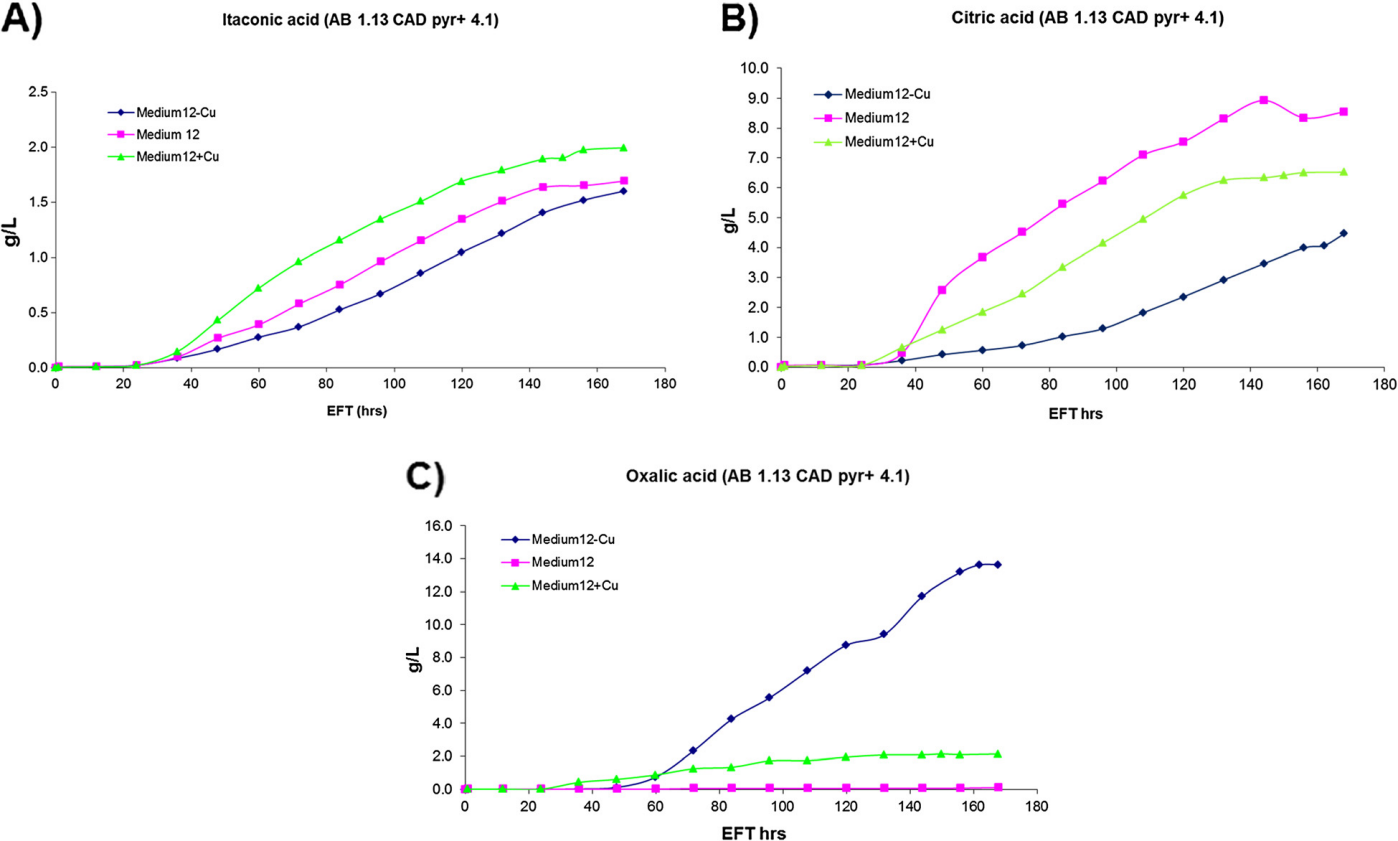
**Itaconic-, citric- and oxalic acid production in fermentation media of *A. niger *.** Organic acid content (itaconic- citric- and oxalic- acid) of *A. niger* strain AB 1.13 CAD pyr + in Medium 12 with varying copper concentration. (♦—Medium12-Cu with 0.005 mM Cu^2+^; ■—Medium12 with 0.01 mM Cu^2+^ ; ▲—Medium12 + Cu with 0.02 mM Cu^2+^) (EFT: Electronic Fermentation Time).

**Figure 4:**
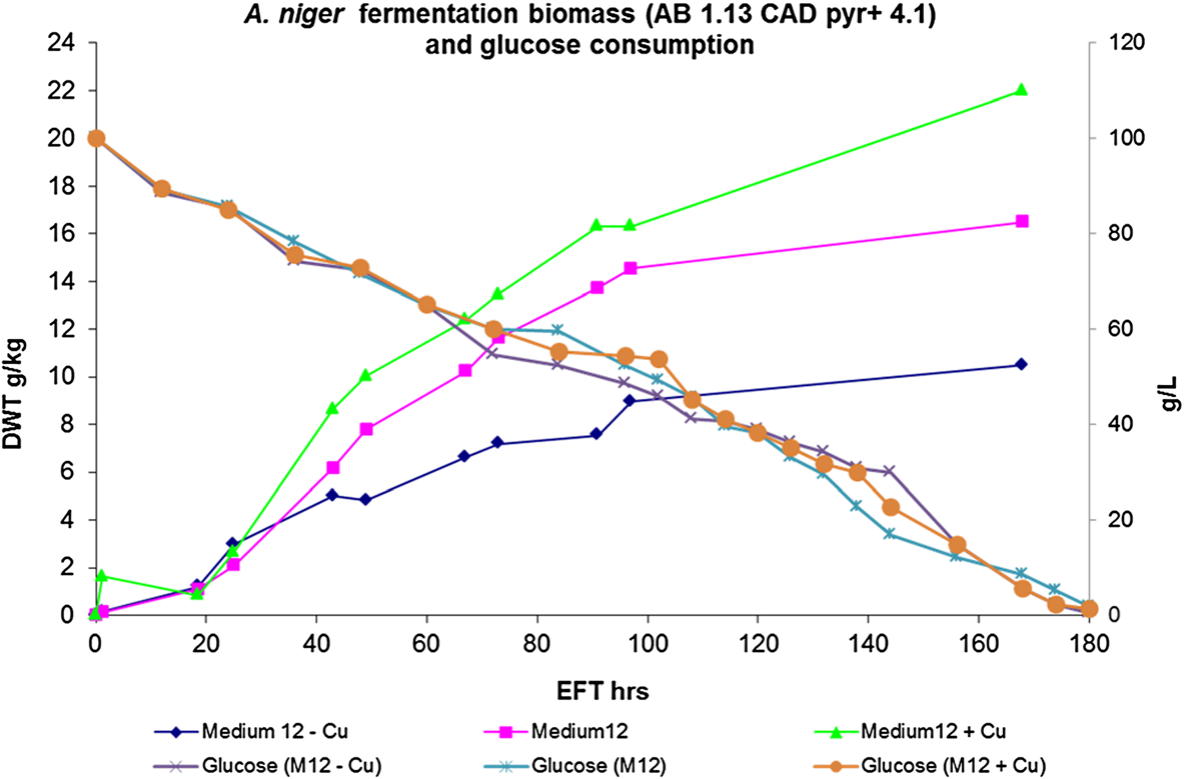
**Biomass growth and glucose consumption of *A. niger *in fermentations with varying copper concentrations.** Biomass determination of mycelium in batch fermentation (♦—Medium12-Cu with 0.005 mM Cu^2+^; ■—Medium12 with 0.01 mM Cu^2+^ ; ▴—Medium12 + Cu with 0.02 mM Cu^2+^). The glucose consumption of three batch fermentations were shown on the secondary Axis (**X**— Medium12-Cu with 0.005 mM Cu^2+^; **□**—Medium12 with 0.01 mM Cu^2+^ ; ●—Medium12 + Cu with 0.02 mM Cu^2+^).

The levels of itaconic acid, citric acid and oxalic acid of *A. niger* transformant AB 1.13 CAD pyr + 4.1 in medium 12 containing three concentrations of copper were analyzed. As presented in Figure 3A, the production rate of itaconic acid is positively correlated with the copper concentration while this correlation is different for citric acid (Figure 3B) and oxalic acid (Figure 3C). In the low-copper medium (0.005 mM Cu^2+^), high levels of oxalic acid are accumulated whereas the highest amount of citric acid is produced in the medium-copper medium. In addition to organic acids, we determined biomass and glucose consumption. Glucose consumption of *A. niger* strain AB 1.13 CAD pyr + 4.1 among all three media is nearly the same (Figure 4). Increase in biomass showed a strong positive correlation with copper levels (Figure 4). The three main organic acids were quantified and the average concentrations (g/L) of duplicate measurements are listed in Table 4. As indicated, a two fold increase of the percentage of itaconic acid is obtained in the high-copper medium. (Table 4).

**Table 4:** Itaconic acid levels among the main three organic acids (itaconic-, citric- and oxalic acid) in batch cultivation using different copper concentrations

	**Itaconic acid*g /L***	**Citric acid*g /L***	**Oxalic acid*g /L***	**Itaconic acid percentage from the total organic acids**
**Medium12 - Cu**				
*108 hrs*	0.086	1.774	5.851	10%
*144 hrs*	1.226	3.323	9.722	9%
*168 hrs*	1.453	3.857	11.557	9%
**Medium12**				
*108 hrs*	1.052	6.975	0.050	13%
*144 hrs*	1.490	8.877	0.062	14%
*168 hrs*	1.639	8.923	0.093	15%
**Medium12 + Cu**				
*108 hrs*	1.430	5.128	1.262	18%
*144 hrs*	1.768	6.229	1.453	19%
*168 hrs*	1.945	6.570	1.473	20%

The levels are averages of the duplicate measurements (g/L) (end concentrations deviate less than 5 %).

## Discussion

Shake flask cultures are commonly used for screening of new *A. niger* transformants [10,17]. However, for analysis of large numbers of transformants, this approach is not entirely suitable. Therefore, we used 96-well plates for screening transformants similar to the methods described previously [18,19]. The screening results are consistent since the standard deviations are less than 6 %.

As suggested in our previous study we introduced extra copies of *gpdA* in *A. niger* strain CAD 10.1 in order to improve itaconic acid production via molecular modification [4]. In addition, based on the research of the hemoglobin domain in *A terreus*[7] and *A. oryzae*[20], extra copies of *hbd1* were introduced in *A. niger* strain CAD 10.1 to increase the production level of itaconic acid. Co-transformation resulted in strains with increased itaconic acid levels compared to the parental strain. However, comparison to pyrG + complemented transformants showed that the increase was the results of complementation of the uridine deficiency alone. Surprisingly, a *gpdA* transformant produced only half amount of itaconic acid in comparison to the parental strain. However, more detailed analysis of this strain in controlled fermentation did not show significant differences with the control strain. This may indicate that the observed effect is specific for plate conditions. Although this shows that *gpdA* expression and itaconic acid production were related, forced over-expression in *A. niger* did not result in increased itaconic acid levels without extending the cultivation period.

Based on the various itaconic acid and citric acid production media described in literature (Table 2), we designed 20 media by modifying the amount of various components in reference medium M1 (Table 1). Medium 8, 12 and 20 were shown to have a positive effect on itaconic acid production while medium 11 and 13 had a negative effect (Figure 2A). Nevertheless, all of these five media had high glucose concentration of 10 %, which had been identified as an important condition for producing organic acids [13,21]. The positive effects were observed with extra Cu^2+^ (M12) or phosphorus (M8), or replacing Na^+^ and Ca^2+^ with K^+^ (M20). Lack of Cu^2+^ (M11) or increment of Fe^2+^ (M13) were shown to result in decreased itaconic acid accumulation. The effect of phosphorous to organic acids production has not yet been demonstrated, except for a positive trend seen due to its limitation in the used medium [22]. Similarly, the effect of K^+^ has also not been documented previously.

Since detailed analysis on trace metal concentration in *A. niger* media showed that citric acid production could be increased by combining low levels of Mn^2+^ with high levels of Fe^2+^[11], and because Fe^2+^ is a cofactor of hemoglobin to bind oxygen, we investigated this combination using medium 13. This medium contained a high concentration of Fe^2+^ and no Mn^2+^. However, neither the itaconic acid nor the citric acid level was improved in our 96-well screen. So, probably under limited oxygen supply in this micro-titer cultivation condition, the oxygen uptake in medium 13 had not been improved compared to medium 1.

Unexpectedly, itaconic acid production was not further improved by combining the maximal value of the positive components of M8, 12, 20 in one medium. In addition, the media yielding improved itaconic acid production were not the same as the ones for citric acid.

Based on the results of our micro-titer plate screenings, we studied the influence of copper in the batch cultivation of *Aspergillus niger* strain AB 1.13 CAD pyr + 4.1. Itaconic acid production level was proved to be positively related with copper concentration in the medium. Moreover, the best itaconic acid producing condition was medium 12 with 0.02 mM Cu^2+^, which yielded a 2.5 folds increase in itaconic acid production compared to the reference medium (Figure 3) [4]. Although the percentage of itaconic acid (20 %) among the total organic acids is lower in comparison to our previous study (80 %), the production level is two folds higher. In this respect, it was interesting to note that *A. niger* was the most copper tolerant among 11 fungal species especially under low pH conditions [23], allowing the use of copper for enhancing itaconic acid production in this species. In our cultivations, copper increment had no toxic effect on biomass growth. Besides, copper ions could increase the production of citric acid as well. The research group of Haq found 0.015 mM of Cu^2+^ could increase the productivity of citric acid by *A. niger*[24,25]. Similarly in our research with *A. niger* strain AB 1.13 CAD pyr + 4.1, highest citric acid production was observed using 0.01 mM Cu^2+^.

Although our current itaconic acid production level (2 g/L) is clearly lower than the levels reached using *A. terreus*, we improved the level with two folds compared to our previous study. As illustrated here, medium components influence itaconic acid production levels by *A. niger*. The fermentation results also indicate that optimal citric acid production conditions were different from optimal itaconic acid production conditions. Our opinion is that the itaconic acid production using *A. niger* is very promising, in particular if high yielding citric acid producing strains would be used [12]. As currently this strain produces more citric acid and oxalic acid than itaconic acid, there is opportunity to further improve itaconic acid production by eliminating oxalic acid [26] and a better conversion of citric acid. Moreover, additional process parameters such as temperature, pH, and dissolved oxygen tension could also be explored. Furthermore, to obtain optimal product formation, more elaborate research might be required based on our initial transcriptomics research [4].

## Conclusions

In this study, itaconic acid production level has been significantly improved. We conclude that micro-titer plate cultivation is suitable for screening large numbers of fungal strains or comparison of various media. Copper is shown to be positively correlated with improved itaconic acid production. Although the role of copper in the metabolic pathway of organic acids is not yet clear, this new insight will allow further medium optimization [4]. The performance of *gpdA* and *hbd1* transformants in itaconic acid production was not better than the non-transformed control. The optimal conditions for itaconic acid production are clearly different from those optimal for citric acid or oxalic acid production. Further research is required for itaconic acid production improvement.

## Competing interests

The authors declare that they have no competing interests.

## Authors’ contributions

AL carried out the strain modification, medium study in the controlled batch cultivation, metabolite analysis and drafted the manuscript. NP participated in the strain modification, medium design and screening. RZ joined the medium study in controlled batch cultivation and related metabolite analysis. PP supervised the study and approved the final manuscript. All authors read approved the final manuscript.

## Author details

^1^TNO Microbiology and Systems biology, PO Box 360, 3700 AJ Zeist, The Netherlands. ^2^Molecular Microbiology & Biotechnology Leiden University, Universiteit Leiden, Postbus 9500, 2300 RA Leiden, Netherlands.
